# Producing High-Quality Buckwheat Sprouts: The Combined Effects of Melatonin and UV-B Treatment

**DOI:** 10.3390/foods15030422

**Published:** 2026-01-24

**Authors:** Xin Tian, Meixia Hu, Weiming Fang, Yongqi Yin

**Affiliations:** 1 College of Food Science and Engineering, Yangzhou University, Yangzhou 210095, China; dx120230241@stu.yzu.edu.cn (X.T.);; 2 Dandong Technician College, Dandong 118000, China

**Keywords:** melatonin, ultraviolet-B, buckwheat, secondary metabolites, gene expression, antioxidant system

## Abstract

Our prior research revealed that UV-B stress enhances bioactive compounds’ biosynthesis in buckwheat sprouts while simultaneously increasing oxidative damage. To address this, we searched for an exogenous hormone capable of promoting bioactive compound accumulation while mitigating UV-B-induced oxidative damage. This study investigated the regulatory effects of exogenous melatonin (MT) on secondary metabolite accumulation and antioxidant systems in buckwheat sprouts under UV-B stress. MT (30 μM) treatment significantly increased the contents of bioactive compounds (flavonoids and total phenolics) in buckwheat sprouts under UV-B stress. Moreover, these contents peaked in 3-day-old sprouts, showing increases of 23.1% and 13.6%, respectively, compared to UV-B-treated. Concurrently, combined UV-B and MT treatment significantly elevated key enzyme activities in the phenylpropanoid pathway and upregulated the related gene expression levels. Additionally, exogenous MT significantly enhanced the antioxidant capacity of sprouts under 3-day UV-B stress, increasing DPPH radical scavenging rate and FRAP values by 8.38% and 12.2%, respectively. MT treatment also upregulated superoxide dismutase activity (32.1%), peroxidase activity (10.3%), and catalase activity (27.2%). It further enhanced the expression of antioxidant-related genes. Collectively, these effects reduced the accumulation of malondialdehyde, hydrogen peroxide, and superoxide anions, thereby mitigating UV-B-induced oxidative damage in sprouts. This research suggests a potential strategy for the targeted enhancement of bioactive compounds in buckwheat sprouts.

## 1. Introduction

Buckwheat (*Fagopyrum esculentum*) is classified within the genus *Fagopyrum*, family *Polygonaceae*. It is an ancient and highly valuable food crop and is widely cultivated worldwide [1,2,3]. Buckwheat is rich in abundant secondary metabolites, especially total phenolics and flavonoids [4]. These secondary metabolites confer many unique benefits to buckwheat. Long-term intake of buckwheat and its related products can effectively reduce blood lipids [5], lower blood glucose levels [6], and enhance antioxidant capacity [7]. In recent years, the concept of “food as medicine” [8] has been widely recognized. It has significantly driven the rapid development of the functional food industry that uses medicinal food ingredients. Buckwheat, as one of the most representative raw materials in this field [9], has seen its related products attract considerable attention. Meanwhile, functional foods and dietary supplements centered on buckwheat secondary metabolites have been launched and gained widespread popularity [10,11,12,13]. This market trend has further stimulated a surge of research focused on strategies to enhance the content of bioactive components in buckwheat [14,15].

Germination is an intrinsic metabolic pathway of plants. It notably increases the content of various bioactive components in plants [16,17,18]. Thus, germination is an effective approach to enhance plant-derived active substances. Existing experimental evidence indicates that ultraviolet irradiation [19,20] and modification of inducing factors [21,22,23,24,25,26,27] can significantly promote the biosynthesis and accumulation of secondary metabolites in plant sprouts. Previous research [28] demonstrated that while UV-B radiation increased total phenolic and flavonoid contents by 24.1% and 18.7%, respectively, in 3-day-old buckwheat sprouts, it simultaneously substantially elevated malondialdehyde (MDA) and hydrogen peroxide (H_2_O_2_) levels in the sprouts, thereby inhibiting sprout growth. Therefore, a critical and pressing challenge remains in formulating strategies that simultaneously enhance secondary metabolite biosynthesis without adversely affecting the normal growth and development of the sprouts.

Melatonin (MT) is an indoleamine compound widely distributed in organisms. As a novel plant hormone, it exerts multiple key functions in plant growth and development [29,30,31]. Studies have shown that exogenous MT application enhances secondary metabolite biosynthesis by activating phenylalanine ammonia-lyase (EC 4.3.1.5, PAL) and cinnamate 4-hydroxylase (EC 1.14.13.11, C4H) activity [32], and upregulating the transcription of flavonoid biosynthetic genes such as *chalcone Synthase* (EC 2.3.1.74, CHS) and *chalcone isomerase* (EC 5.5.1.6, CHI) [33]. Notably, Zu et al. [34] reported that exogenous MT application significantly enhanced flavonoid biosynthetic efficiency in *Ginkgo biloba*. This effect was achieved by upregulating the expression of flavonoid synthesis-related structural genes and modulating regulatory transcription factors, such as members of the ZIP, HLH, and MYB families.

Furthermore, exogenous MT enhances plant resilience by modulating stress response mechanisms, primarily through two pathways [35,36,37,38,39]. Firstly, MT mitigates oxidative stress by upregulating antioxidant enzyme systems essential for reactive oxygen species (ROS) scavenging. Secondly, it functions synergistically with other antioxidants to bolster oxidative defense. Exogenous MT mitigates the inhibitory effects of sodium carbonate on the growth of maize sprouts [40]. It also enhances maize tolerance to abiotic stresses by increasing antioxidant enzyme activities and reducing H_2_O_2_ and MDA levels [41]. Recent studies further confirm that exogenous MT simultaneously enhances antioxidant defense, alleviates oxidative damage, and stimulates secondary metabolite biosynthesis [42,43,44]. Consistent with this, our previous research showed that exogenous MT increases total phenolics and flavonoids in UV-B-stressed buckwheat sprouts while promoting their growth. However, the regulatory mechanisms underlying MT-mediated modulation of secondary metabolite biosynthesis and oxidative stress resistance in UV-B-exposed buckwheat sprouts remain elusive. Therefore, it is of great necessity and innovation to carry out studies on the effect of combined UV-B and MT treatment on the content of secondary metabolites in buckwheat.

This study aims to investigate the effects of exogenous MT on secondary metabolite content and oxidative systems in buckwheat sprouts under UV-B stress. By measuring and analyzing physiological and biochemical parameters, enzyme activities, and gene expression levels in buckwheat sprouts under different treatments, the study reveals the mechanisms by which exogenous MT regulates secondary metabolite content and oxidative systems in UV-B-stressed buckwheat sprouts. This study provides technical insights that can support strategies for enhanced bioactive compound production.

## 2. Materials and Methods

### 2.1. Treatment

After 10 min in a 1% sodium hypochlorite solution, rinse the buckwheat seeds thoroughly with deionized water until the solution is neutral. Subsequently, soak in deionized water at 25 °C for 12 h. Transfer the seeds onto a germination tray lined with gauze and incubate within a controlled germination chamber maintained at 25 °C.

Treatment details:

(1) UV-B stress: A narrowband UV-B lamp (30 µmol m^−2^ s^−1^, central wavelength 313 nm, 20 W, UVB-313, 290 nm–320 nm, Guangzhou Longpro Electric, Inc., Guangzhou, China) was used during germination with an 8/16 h light/dark cycle, and 30 mL of deionized water was sprayed every 12 h. (2) MT: An LED white light bulb (Guangzhou Longpro Electric, Inc., Guangzhou, China) was used during germination according to a cycle of 8/16 h light/dark and spray 30 mL of 30 μM MT (Hunan Vokai Biotechnology Co., Ltd., Hunan, China) solution every 12 h. For MT dissolution, first dissolve it in a small amount of ethanol (Hunan Vokai Biotechnology Co., Ltd., Hunan, China), then dilute with deionized water to the desired volume. (3) Combined UV-B and MT treatment: 8 h/day UV-B irradiation and application of a 30μM MT solution every 12 h. These treatments were labeled U, M, and UM. At specific germination stages (1, 3, and 5 days), uniformly developed sprouts were randomly selected from each treatment, rinsed with deionized water, and stored at −80 °C for subsequent parameter measurements.

### 2.2. Physiological Metabolism

For lipid peroxidation assessment, tissue homogenates were prepared in 10% trichloroacetic acid (TCA, Shanghai Hushi Chemical Co., Ltd., Shanghai, China) containing 0.65% thiobarbituric acid (TBA, Shanghai Hushi Chemical Co., Ltd., Shanghai, China). The samples were heated at 100 °C for 15 min to facilitate the formation of MDA-TBA adducts. MDA levels were indirectly quantified by measuring absorbance at 535 nm, subtracting the TBA-reacted sample’s absorbance from that of the unreacted plant extract.

H_2_O_2_ content was determined by reacting the supernatant with titanium sulfate (Shanghai Hushi Chemical Co., Ltd., Shanghai, China) reagent, followed by centrifugation to isolate the precipitate. The precipitate was dissolved in sulfuric acid (Shanghai Hushi Chemical Co., Ltd., Shanghai, China), and absorbance was measured at 415 nm, with H_2_O_2_ concentrations extrapolated from a standard curve.

Superoxide anion $\left(O_{2}^{–•}\right)$ content was assessed by homogenizing the sample in phosphate-buffered saline (Shanghai Hushi Chemical Co., Ltd., Shanghai, China), centrifuging to obtain the supernatant, which was then reactively incubated with hydroxylamine hydrochloride (Shanghai Hushi Chemical Co., Ltd., Shanghai, China) for one hour. Subsequent measurement of absorbance at 530 nm allowed for $O_{2}^{–•}$ quantification via a calibration curve generated using sodium nitrite (Shanghai Hushi Chemical Co., Ltd., Shanghai, China) as the standard.

### 2.3. Total Phenolics and Flavonoids Contents

The total phenolics and flavonoids contents in the sprouts were determined according to the method of Tian et al. [28]. Briefly, the 0.2 g sprouts were subjected to extraction using 50% (*v*/*v*) methanol (Shanghai Hushi Chemical Co., Ltd., Shanghai, China). The extraction mixture was then centrifuged at a force of 10,000× *g* for a duration of 15 min to separate the soluble compounds. The resulting supernatant, measuring 1 mL, was carefully combined with 1.0 mL of 0.2 mM Folin phenol (Shanghai Hushi Chemical Co., Ltd., Shanghai, China) reagent and 2.0 mL of 2% (*w*/*v*) sodium carbonate (Shanghai Hushi Chemical Co., Ltd., Shanghai, China). This mixture was allowed to react for 2 h in darkness to prevent photo-degradation. The total phenolic content was subsequently quantified by measuring the absorbance at a wavelength of 765 nm (UV spectrophotometer). For calibration purposes, gallic acid (Hunan Vokai Biotechnology Co., Ltd., Hunan, China) served as the standard reference compound.

The samples were collected and homogenized in methanol. The resultant homogenate was subjected to centrifugation, and the supernatant was harvested subsequently. The supernatant was mixed sequentially with sodium nitrite (Hunan Vokai Biotechnology Co., Ltd., Hunan, China), aluminum nitrate (Hunan Vokai Biotechnology Co., Ltd., Hunan, China), and sodium hydroxide (Hunan Vokai Biotechnology Co., Ltd., Hunan, China). Following a 15 min incubation, the absorbance value was detected at a wavelength of 510 nm. A standard curve was constructed using rutin (Hunan Vokai Biotechnology Co., Ltd., Hunan, China) as the reference standard, and the flavonoids content in the samples was calculated based on this curve.

Flavonoidscontent(μg/sprout)=C×V×n/30

In the formula, C refers to the rutin concentration (mg/mL) derived from the standard curve, V stands for the total volume (mL) of the sample extract, and n represents the dilution factor.

### 2.4. Antioxidant Capacity

Buckwheat sprouts were homogenized with 80% methanol, centrifuged at 10,000× *g* for 15 min, and the supernatant was used as the test solution. In detail, 1,1-diphenyl-2-trinitrophenylhydrazine (DPPH, TargetMol Chemicals Inc., Boston, USA) working solution: 0.1 mmol/L, prepared in 95% ethanol. 2,2′-azino-bis 3-ethylbenzothiazoline-6-sulfonic acid (ABTS, Hunan Vokai Biotechnology Co., Ltd., Hunan, China) working solution: 7 mmol/L ABTS solution mixed with 2.45 mmol/L potassium persulfate (Hunan Vokai Biotechnology Co., Ltd., Hunan, China) (1:1, *v*/*v*) and incubated in the dark for 12–16 h, then diluted with phosphate-buffered saline (PBS, 0.01 mol/L, pH 7.4) to an absorbance of 0.70 ± 0.02 at 734 nm. A volume of 0.1 mL supernatant was mixed with 2.9 mL DPPH solution. The mixture was incubated in the dark for 30 min, after which the absorbance at 517 nm was measured using a UV spectrophotometer (DR6000, Shanghai Ruishi Technology Co., Shanghai, China). The antioxidant capacity was expressed as DPPH clearance rate (%). A total of 0.1 mL supernatant was combined with 2.9 mL ABTS working solution. After 30 min of dark incubation, the absorbance of the mixture at 734 nm was determined with a UV spectrophotometer, and the result was calculated as ABTS clearance rate (%).

Then, 0.25 mL supernatant, 1.0 mL PBS, and 1.0 mL potassium ferricyanide (Hunan Vokai Biotechnology Co., Ltd., Hunan, China) solution were mixed and incubated. Subsequently, 1.0 mL of 10% TCA was added to the mixture and vortexed thoroughly. The absorbance of the resulting mixture was measured at 700 nm using a UV spectrophotometer, and the antioxidant activity was reported as FRAP clearance rate (%).

### 2.5. Antioxidant Enzyme Activity

The activities of antioxidant enzymes were determined according to Tian et al. [28]. The activity units (U) of ascorbate peroxidase (EC 1.11.1.11, APX) and peroxidase (EC 1.11.1.7, POD) are defined as a change in absorbance of 0.01 per minute per gram of fresh sample at wavelengths of 290 nm and 470 nm, respectively. The U of superoxide dismutase (EC 1.15.1.1, SOD) is established as a change in absorbance of 0.02 per minute per gram of fresh sample at a wavelength of 560 nm. The U of catalase (EC 1.11.1.6, CAT) is defined as a change in absorbance of 0.1 per minute per gram of fresh sample at a wavelength of 240 nm.

### 2.6. Synthase Enzyme Activity

Buckwheat sprout samples were homogenized using a Tris-HCl buffer solution, adjusted to a pH of 8.9 and a concentration of 0.1 M. The homogenate was then subjected to centrifugation at 12,000× *g* for 15 min at 4 °C to isolate the supernatant containing the target enzymatic activities. Enzyme activity units for phenylalanine ammonia-lyase (EC 4.3.1.5, PAL), cinnamate 4-hydroxylase (EC 1.14.13.11, C4H), and 4-coumarate-CoA ligase (EC 6.2.1.12, 4CL) were defined as the enzymatic catalysis resulting in a change of 0.01 optical density units per minute at 290 nm, 340 nm, and 333 nm, respectively, measured with a UV spectrophotometer.

The supernatant derived from the preceding extraction procedure was employed as a crude enzymatic extract. A precise volume of 0.75 mL of this crude enzyme solution was introduced into an assay mixture comprising 2 mL of 0.05 M Tris-HCl buffer (pH 7.4), supplemented with 7.5 mg/mL bovine serum albumin (Hunan Vokai Biotechnology Co., Ltd., Hunan, China), 50 mM potassium cyanide (Hunan Vokai Biotechnology Co., Ltd., Hunan, China), and 50 µL of a 1 mg/mL hydroxylated chalcone (Hunan Vokai Biotechnology Co., Ltd., Hunan, China) solution. The enzymatic activity assay was conducted under standardized conditions at 30 °C for 30 min. The activity of chalcone isomerase (EC 5.5.1.1, CHI) was quantified by measuring changes in absorbance at 381 nm using a UV spectrophotometer.

### 2.7. Gene Expression Levels

Total RNA was extracted from buckwheat sprouts using an RNA extraction kit (RC411, Vazyme, Nanjing, China). The A_260_/A_280_ ratio was measured with a UV spectrophotometer to ensure RNA purity (1.8–2.0 is acceptable). Qualified total RNA served as a template for cDNA synthesis using a reverse transcription kit (R423, Vazyme). Quantitative real-time PCR (qRT-PCR) was performed on the CFX96 Touch Real-Time PCR Detection System (Bio-Rad) using the SYBR Green Kit (RR820A, Vazyme). The gene of *FeActin* from buckwheat sprouts was selected as the standardized reference gene. Primer pairs for both target and reference genes were designed using Primer Premier 6.0 software. All standard curves demonstrated amplification efficiencies within 95–105% and R^2^ values > 0.99. Following qPCR reactions, melting curve analysis for each primer pair confirmed the presence of a single melting peak, verifying amplification specificity. Using cDNA as a template, qRT-PCR kits were employed to determine the relative expression levels of genes associated with the antioxidant system, phenylpropanoid metabolism, and transcription factors. The relative expression levels of each gene were calculated using the 2^−ΔΔCt^ method, with three technical replicates per sample. Primer sequences are listed in Table S1.

### 2.8. Statistical Analysis

All experiments were conducted in triplicate, with data reported as mean ± standard deviation (SD). Statistical analysis employed one-way ANOVA and Tukey’s multiple comparisons test, with significance thresholds set at *p* < 0.05.

## 3. Results

### 3.1. Total Phenolics and Flavonoids Contents

**Figure 1:**
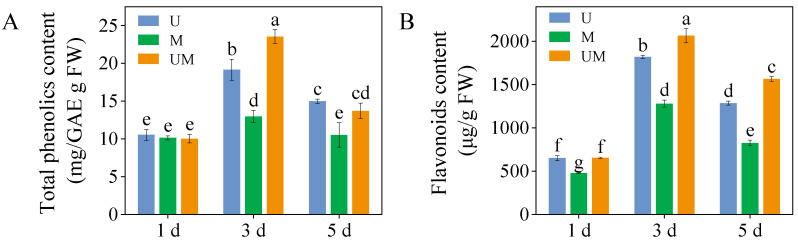
Effect of exogenous MT on total phenolics content (**A**) and flavonoids content (**B**) in buckwheat sprouts under UV-B stress. Different lowercase letters indicate significant differences between groups (*p* < 0.05). U: 8 h UV-B irradiation. M: 30 μM MT. UM: 8 h UV-B irradiation combined with 30 μM MT treatment. *n* = 3 biological replicates.

On day three of germination, comparative analysis revealed that exogenous MT significantly elevated total phenolic in sprouts under UV-B stress, with a 23.1% increase relative to UV-B treatment alone (Figure 1A, *p* < 0.05). Additionally, exogenous MT markedly enhanced flavonoids content compared to UV-B stress, demonstrating respective increases of 13.6% and 21.8% (Figure 1B).

### 3.2. The Contents of H_2_O_2_, MDA, and $O_{2}^{-•}$

**Figure 2:**
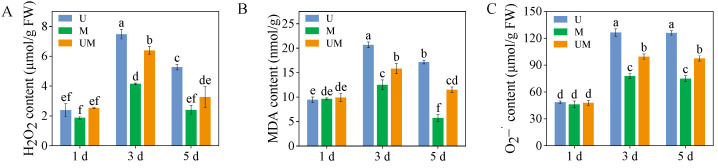
Effect of exogenous MT on H_2_O_2_ (**A**), MDA (**B**), and $O_{2}^{–•}$ (**C**) content in buckwheat sprouts under UV-B stress. Different lowercase letters indicate significant differences between groups (*p* < 0.05). U: 8 h UV-B irradiation. M: 30 μM MT. UM: 8 h UV-B irradiation combined with 30 μM MT treatment. *n* = 3 biological replicates.

There were no significant differences observed in H_2_O_2_, MDA, and $O_{2}^{–•}$ contents among treatments on the first day (Figure 2, *p* > 0.05). Compared with UV-B stress, exogenous MT treatment significantly reduced H_2_O_2_, MDA, and $O_{2}^{–•}$ content in 3-day-old and 5-day-old sprouts. Specifically, exogenous MT significantly decreased H_2_O_2_, MDA, and $\;O_{2}^{–•}$ contents in 3-day-old sprouts by 14.7%, 23.5%, and 21.4%, respectively. These results indicated that exogenous MT effectively alleviates oxidative damage caused by UV-B stress in buckwheat sprouts.

### 3.3. Antioxidant Enzyme Activity

**Figure 3:**
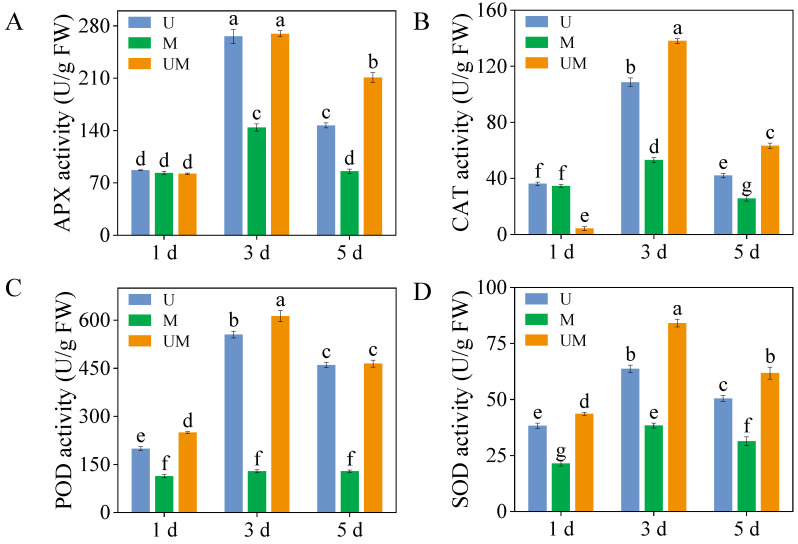
Effect of exogenous MT on activities of antioxidant enzymes ((**A**): APX activity, (**B**): CAT activity, (**C**): POD activity, and (**D**): SOD activity) in buckwheat sprouts under UV-B stress. Different lowercase letters indicate significant differences between groups (*p* < 0.05). U: 8 h UV-B irradiation. M: 30 μM MT. UM: 8 h UV-B irradiation combined with 30 μM MT treatment. *n* = 3 biological replicates.

In 3-day-old sprouts, APX activity peaked under combined UV-B and MT treatments, with values of 265.8 U/g and 269.6 U/g, respectively (Figure 3A). Moreover, in 5-day-old sprouts, exogenous MT significantly increased APX activity compared to UV-B stress, reaching 1.43 times the APX activity in UV-B-treated sprouts (Figure 3A, *p* < 0.05). Similarly, exogenous MT significantly increased CAT activity in sprouts during germination compared to UV-B treatment, reaching 1.19-, 1.31-, and 1.51-fold levels relative to UV-B treatment (Figure 3B). However, POD activity in sprouts showed no significant changes under MT treatment alone at any germination stage. However, exogenous MT application significantly increased POD activity in 1-day-old and 3-day-old sprouts. POD activity reached a maximum of 612.75 U/g after 3 days of combined UV-B and MT treatment (Figure 3C). SOD activity in 3-day-old sprouts was the highest across all treatments, reaching 63.74 U/g, 38.38 U/g, and 84.07 U/g, respectively (Figure 3D). Furthermore, compared to UV-B stress, the addition of exogenous MT significantly increased SOD activity in sprouts, with increases of 13.86%, 31.92%, and 22.31%, respectively.

### 3.4. Antioxidant Capacities

**Figure 4:**
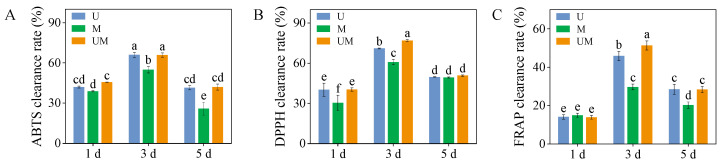
Effect of exogenous MT on antioxidant capacities ((**A**): ABTS clearance rate, (**B**): DPPH clearance rate, and (**C**): FRAP clearance rate) in buckwheat sprouts under UV-B stress. Different lowercase letters indicate significant differences between groups (*p* < 0.05). U: 8 h UV-B irradiation. M: 30 μM MT. UM: 8 h UV-B irradiation combined with 30 μM MT treatment. *n* = 3 biological replicates.

Across all treatment groups, ABTS, DPPH, and FRAP scavenging rates peaked in 3-day-old sprouts. Notably, MT substantially elevated the DPPH and FRAP clearance capacities of 3-day-old sprouts exposed to UV-B stress, with respective increments of 8.37% and 12.11%. Moreover, relative to MT treatment, the combined UV-B and MT treatment significantly enhanced the ABTS and DPPH scavenging rates in 1-day-old sprouts, as well as the ABTS, DPPH, and FRAP radical scavenging rates in 3-day-old and 5-day-old sprouts (Figure 4).

### 3.5. Critical Enzyme Activities

**Figure 5:**
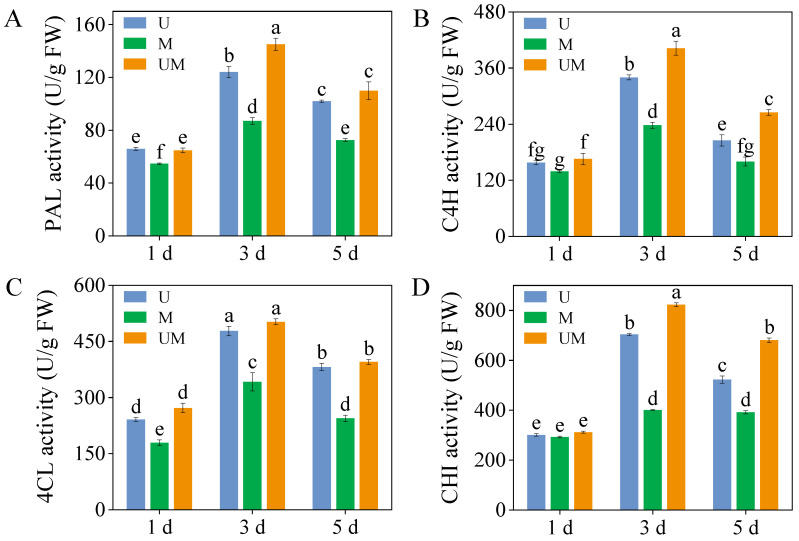
Effect of exogenous MT on activities of critical enzymes ((**A**): PAL activity, (**B**): C4H activity, (**C**): 4CL activity, and (**D**): CHI activity) in buckwheat sprouts under UV-B stress. Different lowercase letters indicate significant differences between groups (*p* < 0.05). U: 8 h UV-B irradiation. M: 30 μM MT. UM: 8 h UV-B irradiation combined with 30 μM MT treatment. *n* = 3 biological replicates.

Although the MT had no significant effect on PAL, C4H, 4Cl, and CHI activities in sprouts under UV-B stress on 1-day-old sprouts, it significantly increased PAL, C4H, and CHI activities in 3-day-old sprouts (Figure 5A,B,D, *p* < 0.05). Under combined UV-B and MT treatment, PAL, C4H, and CHI activities in 3-day-old sprouts peaked at 145.10 U/g, 402.55 U/g, and 822.47 U/g, respectively. These values represented increases of 16.89%, 18.34%, and 16.99%, respectively, compared to the UV-B-treated. Furthermore, in 5-day-old sprouts, exogenous MT combined with UV-B treatment resulted in C4H and CHI activities that were 1.29-fold and 1.30-fold higher, respectively, than those observed under UV-B stress.

### 3.6. Relative Expression Levels

Compared with UV-B treatment, MT significantly upregulated the expression levels of *FeAPX*, *FePOD*, and *FeSOD* in 3-day-old sprouts. *FeSOD* expression level was most prominently regulated, reaching 2.6-fold that of sprouts exposed to UV-B treatment. Concurrently, exogenous MT markedly increased *FeAPX* and *FeSOD* expression levels in 5-day-old sprouts. Furthermore, across all treatment groups, 3-day-old sprouts exhibited significantly higher expression levels of *FePAL*, *FeC4H*, *Fe4CL*, *FeCHS*, *FeCHI*, and *FeF3H* compared to 1-day-old sprouts. Notably, the expression level of *FeCHI* was most strongly induced by this combined intervention. With the increase in germination time, the expression levels of *FeTCP15* and *FeMYB11* showed an increasing trend under UV-B treatment and combined UV-B and MT treatment. In contrast, the expression levels of *FeTCP15* and *FeMYB11* showed no significant changes in MT-treated sprouts.

## 4. Discussion

Total phenolics and flavonoids are recognized natural functional components with multiple physiological benefits, including scavenging free radicals, anti-inflammatory effects, and metabolic regulation [7]. Enhancing their content significantly boosts the nutritional value of buckwheat sprouts [45,46], meeting consumer demand for health foods and improving product market competitiveness. UV-B stress is a typical abiotic stress factor in both agricultural production and natural environments. It can induce the biosynthesis of plant secondary metabolites by activating related signal transduction pathways and metabolic regulatory networks [47]. Previous studies have confirmed that UV-B stress significantly increases the biosynthesis of secondary metabolites such as phenolics and flavonoids in buckwheat sprouts. However, it also causes excessive accumulation of ROS, thereby elevating oxidative damage in the sprouts [28,47]. As a novel plant hormone, MT not only enhances secondary metabolite synthesis but also mitigates oxidative damage caused by abiotic stresses [42,43,44]. This study similarly found that exogenous MT application significantly boosted total phenolic and flavonoids biosynthesis while alleviating UV-B-induced oxidative damage in buckwheat sprouts.

Oxidative damage caused by excessive accumulation of ROS is one of the key physiological responses in plants to abiotic stress. ROS such as H_2_O_2_ and O_2_^−^· can attack biological macromolecules such as proteins, lipids, and nucleic acids in plant cells. This attack induces cell membrane damage, enzyme inactivation, and genetic material impairment, ultimately compromising plant growth and development [48]. Activating and enhancing the antioxidant system within plants is a crucial strategy for defending against this damage [49]. The antioxidative capacity of buckwheat sprouts fundamentally relies on the combined interaction between enzymatic and non-enzymatic antioxidant mechanisms. The enzymatic antioxidant system mainly includes SOD, POD, CAT, APX, and other antioxidant enzymes, which work synergistically to scavenge different types of ROS. The non-enzymatic antioxidant system is composed of phenolics, flavonoids, ascorbic acid, glutathione, and other substances with antioxidant activity [50]. As a key regulatory substance in plant stress responses, MT not only directly scavenges ROS as an antioxidant within plant cells but also indirectly promotes antioxidant enzyme activity, inhibits pro-oxidant enzyme activity, and increases the content of non-enzymatic antioxidants such as phenolic compounds [35,36,37,38,39]. Studies indicate that MT alleviates ROS accumulation by upregulating enzymatic antioxidants, thereby safeguarding plants from oxidative damage induced by abiotic stresses like salinity [51] and drought [52]. For example, MT application significantly enhances the activities of SOD, POD, CAT, and APX in NaCl-stressed maize by upregulating the transcriptional expression of antioxidant enzyme genes. This effectively scavenges excess ROS and alleviates membrane peroxidation damage [44]. Consistent with these findings, our study showed that under UV-B stress, exogenous MT significantly increased the expression levels of antioxidant enzyme genes (*FePOD, FeSOD*, and *FeAPX*) in three-day-old buckwheat sprouts, while markedly enhancing the activities of CAT, POD, and SOD (Figure 3B–D). Further analysis revealed a significant reduction in MDA content (Figure 2B), a product of membrane lipid peroxidation. This indicates that MT effectively preserved the structural integrity of the cell membrane. Concurrently, H_2_O_2_ content (Figure 2A) markedly decreased, suggesting that the ROS scavenging efficiency of buckwheat sprouts was substantially enhanced under MT treatment.

It is noteworthy that the changes in gene expression levels and enzyme activity in this study were not entirely synchronized. For example, the magnitude of change in *FeSOD* gene expression was greater than that of its corresponding enzyme activity. This phenomenon is not unique to our study and likely stems from the spatiotemporal characteristics of gene regulation [53], as well as the post-transcriptional and post-translational modifications of genes [54]. These results further indicated that exogenous MT fortifies the antioxidative defense system in sprouts exposed to UV-B stress by upregulating antioxidant enzyme gene transcription and augmenting enzymatic activity. This molecular response mitigates ROS accumulation and inhibits lipid peroxidation of cellular membranes, thereby enhancing the stress tolerance of sprouts against UV-B stress.

In addition, flavonoids and total phenolics are secondary metabolites in plants with antioxidant capabilities, serving as key components of non-enzymatic antioxidants and forming another line of defense in ROS scavenging. The addition of MT can induce the synthesis of secondary metabolites in plants. For example, MT counteracts the stress effects of ethylene on soybean sprouts by increasing their isoflavone content [43]. In our study, the addition of exogenous MT significantly elevated flavonoids and total phenolic content (Figure 1) in buckwheat sprouts under UV-B stress, and the antioxidant capacity (DPPH, ABTS) exhibited consistent trends with the content of these secondary metabolites. The addition of MT can induce the synthesis of secondary metabolites in plants.

**Figure 6:**
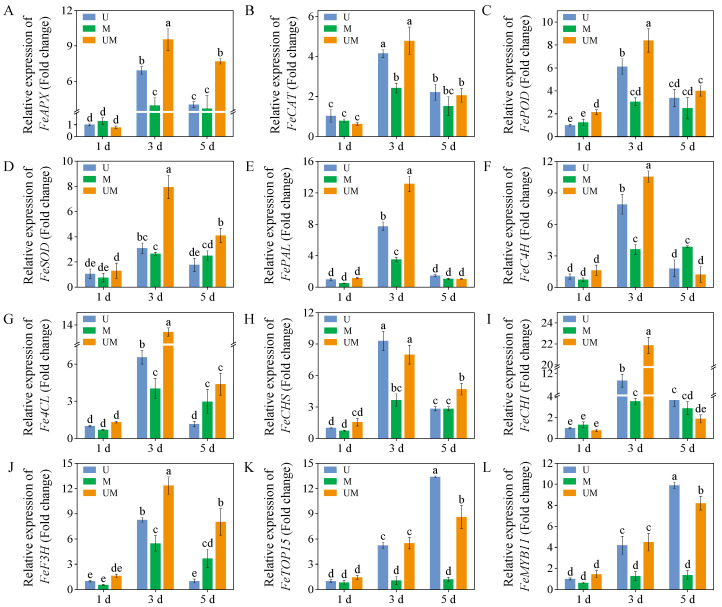
Effects of exogenous MT on relative gene expression levels ((**A**): *FeAPX*, (**B**): *FeCAT*, (**C**): *FePOD*, (**D**): *FeSOD*, (**E**): *FePAL*, (**F**): *FeC4H*, (**G**): *Fe4CL*, (**H**): *FeCHS*, (**I**): *FeCHI*, (**J**): *FeF3H*, (**K**): *FeTOP15*, and (**L**): *FeMYB11*) in buckwheat sprouts under UV-B stress. Relative expression levels of each gene in buckwheat sprouts germinated for 1 day under UV-B treatment were used as the control, with their values set to 1.0. *n* = 3 biological replicates. Different lowercase letters indicate significant differences between groups (*p* < 0.05).

The biosynthesis pathways of total phenolics and flavonoids are derived from the phenylpropanoid metabolic pathway, which constitutes the core metabolic network for plant secondary metabolite synthesis [55]. PAL, the rate-limiting enzyme initiating the phenylpropanoid pathway, catalyzes the deamination of phenylalanine to form cinnamic acid. As a key precursor enzyme, it directly or indirectly participates in the biosynthesis of various secondary metabolites, such as phenolics, flavonoids, and lignins. 4CL functions as a central node enzyme in the phenylpropanoid and flavonoid biosynthetic networks. It catalyzes the conversion of cinnamic acid and its derivatives to coumaroyl-CoA, a critical branch point linking the general phenylpropanoid pathway to specific flavonoid biosynthesis. Thus, 4CL plays an irreplaceable and integral role in flavonoid production. Notably, the enzymatic activities and gene expression levels of these key enzymes collectively modulate the biosynthesis efficiency and accumulation of phenolics and flavonoids, forming a multi-level regulatory module in the metabolic pathway [56]. In this study, exogenous MT was found to enhance the production and accumulation of defensive metabolites such as total phenolics and flavonoids in buckwheat sprouts under UV-B stress (Figure 1), which was closely associated with the induced activities of PAL, C4H, and 4CL (Figure 5A–C). Further evidence showed that the accumulation patterns of these metabolites were highly consistent with the expression patterns of their upstream regulatory genes *FePAL*, *Fe4CL*, and *FeC4H* (Figure 6E–G). This result clearly suggests that exogenous MT can effectively upregulate the transcriptional activity of these essential enzyme genes in the phenylpropanoid biosynthetic pathway, thereby promoting the catalytic efficiency of the corresponding enzymes and ultimately enhancing flavonoid and phenolic production. However, in this study, unlike in 3-day-old sprouts, the FeC4H expression level in 5-day-old MT-treated sprouts was significantly higher than that in UV-B-stressed sprouts. Notably, the corresponding total phenolic and flavonoid contents in these MT-treated sprouts were lower than those in UV-B-stressed sprouts. This result indicates that the biosynthesis of total phenolics and flavonoids is not determined by a single gene or enzyme but depends on the synergistic action of multiple genes, enzymes, and even metabolic pathways [57]. Furthermore, MT treatment significantly elevated the relative expression levels of key biosynthetic genes *FeCHI* and *FeF3H* under UV-B treatment, resulting in higher flavonoid accumulation than in the UV-B-only treatment. These findings indicate that MT actively influences the transcriptional regulation of functional genes in the flavonoid biosynthetic pathway, thereby promoting flavonoid synthesis in buckwheat sprouts.

Beyond directly regulating metabolic enzymes, transcription factors can modulate the biosynthesis of secondary metabolites in response to environmental stress. MYB transcription factors, particularly MYB11, have been well established as key regulators of flavonoid synthesis [58,59]. *MYB11* expression levels’ upregulation directly binds to the promoter regions of structural genes like *CHS* and *CHI*, thereby promoting transcription and activating the flavonoid biosynthetic pathway in tobacco [60]. Concurrently, members of the TCP transcription factor family participate in both plant growth and development and regulate stress responses. They may form transcription factor complexes in synergy with the MYB family to jointly regulate the gene expression levels of metabolic enzymes [61]. This study revealed that under both UV-B stress and MT combined with UV-B treatment, the expression levels of *FeTCP15* and *FeMYB11* genes in buckwheat sprouts showed a significant upward trend with prolonged germination time. In contrast, no obvious changes were observed in the control group treated with MT alone (Figure 6). This differential expression pattern suggests that these two transcription factor families (TCP and MYB) may specifically function as downstream regulators of UV-B stress signals, with their activation dependent on UV-B stress stimulation. Furthermore, the enhanced expression levels of *FeTCP15* and *FeMYB11* in the MT + UV-B group indicate that MT amplifies the stress-induced transcriptional responses mediated by these transcription factors. It is speculated that MT may interact with the UV-B stress signaling pathway to promote the activation of *FeTCP15* and *FeMYB11*, thereby synergistically regulating the upregulation of downstream structural genes (*FeCHI*, *FeF3H*, and *FePAL*) in the flavonoid and phenylpropanoid metabolic pathways. This regulatory cascade mediates the enhancing effects of MT on secondary metabolite biosynthesis and antioxidant activity. These findings not only confirm the regulatory roles of *FeTCP15* and *FeMYB11* in secondary metabolite biosynthesis in buckwheat sprouts but also provide positive evidence for MT’s mechanism in enhancing plant stress resistance.

## 5. Conclusions

This research examined the modulatory influence of exogenous MT on secondary metabolite biosynthesis in buckwheat sprouts under UV-B stress. Findings demonstrated that 30 μM MT enhanced flavonoids’ biosynthesis through significant upregulation of PAL, C4H, 4CL, and CHI enzymatic activities, along with increased transcriptional levels of their respective genes. Additionally, exogenous MT augmented the antioxidant defense system by elevating the activity of antioxidant enzymes and their gene expression, thereby conferring increased tolerance to UV-B-induced oxidative stress. Consequently, the combined application of exogenous MT with UV-B irradiation emerges as a viable approach for biofortification of bioactive compounds in buckwheat.

## Data Availability

The original contributions presented in this study are included in the article/Supplementary Materials. Further inquiries can be directed to the corresponding authors.

## Supplementary Materials

The following supporting information can be downloaded at https://www.mdpi.com/article/10.3390/foods15030422/s1. Table S1: Sequence-specific primers used in the present study.
