# Supplementary material for: Producing High-Quality Buckwheat Sprouts: The Combined Effects of Melatonin and UV-B Treatment
Source: Foods. 2026 Jan 24;15(3):422. doi: 10.3390/foods15030422 (PMC12896635; doi:10.3390/foods15030422)
Supplement: Supplementary file 1 [file foods-15-00422-s001.zip › foods-4029170-supplementary.pdf]

Supplementary Table S1. Sequence-specific primers used in the present study.

Gene	Forward Primer (5’ - 3’)	Reverse Primer (5' - 3')
FeActin	TCGTGAGAAGATGACCCAGA	CCGAGTCCAGCACAATACCT
FePAL	TCTCCAGAAGCCGAAACAAG	AGCCTTGTTCCTGGATACAT
FeC4H	AACACACTACTCTCAGTTGC	ATTGGGTGATCGAGACTCTT
Fe4CL	CTCTTTCACGTCCACGGTTT	GATGATTTGGTGGATGGTGG
FeCHS	CGTCAAGCGTTTCATGATGT	CAAGGCTTGTGTTGACATGG
FeCHI	ACTTTGAGGAATCCGCTGTGAC	AGGGCTTCAACATGGTGATCTGTA
FeF3H	CAAGGCTTGTGTTGACATGG	GACAGTGATCCAGGTCTTGC
FeCAT	GAGTTTGGTTCCCTTGCTT	TTCATACACTTCACTGGCGT
FeAPX	GCTTCTCTTGAGCTTTGCTGT	TCTGTTGGGGAACACCGAGA
FeSOD	ATGGTGCTCCTGACGATG	CCACTGCCCTTCCAATAAT
FePOD	GTTCTGGTTGGGCTTGG	TTGTCCTCGTCTGTTGGTC
FeTCP15	GATAGGCTTGGCTATGATAGGCC	CAAACACAAATCTCGATGTGGGT
FeMYB11	GGTGGTCAATCAGCTCAGCCCA	TCGGTCCTACCTGGGAAGGCGAGC
